# Mitochondrial and nuclear genomics and the emergence of personalized medicine

**DOI:** 10.1186/1479-7364-6-3

**Published:** 2012-07-05

**Authors:** Ryan L Parr, Luis H Martin

**Affiliations:** 1 Mitomics Inc, Thunder Bay, Ontario, P7A 7T1, Canada

**Keywords:** Mitochondrial genomics, Mitogenome, Genomic deletions, Cancerization field, Biosensor, Heteroplasmy

## Abstract

Developing early detection biosensors for disease has been the long‒held goal of the Human Genome Project, but with little success. Conversely, the biological properties of the mitochondrion coupled with the relative simplicity of the mitochondrial genome give this organelle extraordinary functionality as a biosensor and places the field of mitochondrial genomics in a position of strategic advantage to launch significant advances in personalized medicine. Numerous factors make the mitochondrion organelle uniquely suited to be an early detection biosensor with applications in oncology as well as many other aspects of human health and disease. Early detection of disease translates into more effective, less expensive treatments for disease and overall better prognoses for those at greater risk for developing diseases.

## Introduction

*Nascentes Morimur*—from the moment we are born, we begin to die. This Latin phrase succinctly expresses the motivations behind personalized medicine and genomic research. The goal is to detect the earliest indications of disease process initiation within the human body and prevent disease advancement through early treatment. Ideally, this leads to extended human life spans coupled with enhanced quality of life. While the genetic root of many diseases has been known for decades, the ‘‒omics’ revolution starting with the Human Genome Project (HGP) was the flashpoint that began the personalized medicine movement.

Direct medical advances resulting from the HGP have been minimal compared to the expectations of its champions. Most currently available gene‒specific treatments for cancers, novel therapies for inherited disorders, companion diagnostic genetic markers for drug responsiveness, and gene‒based risk assessments for specific diseases are founded on research that predates the HGP. Very little HGP research has made the transition into practical use in the clinical setting [1].

The greatest hindrance has been the sheer expansiveness of the human genome. Consisting of nearly 6 billion base pairs (bp), finding specific and actionable connections with diseases requires filtering through enormous data sets. Scientists are still developing tools to decipher this information, and applications in the clinical setting are not yet a reality, despite the cost of sequencing an entire genome now at about $1,000.

However, there is a branch of genomic research, specifically mitochondrial genomics (mtgenomics), that is poised to begin entering clinical practice, and in some instances, it already has. Though much emphasis was initially placed on the HGP as a paradigm shift in modern medicine, this declaration would more accurately be made of mtgenomics for a number of reasons that will be outlined in this article.

Simply stating that a new technology or field of knowledge represents a paradigm shift in medicine does not justify its adoption into mainstream medical practice. Utility must be shown on a number of levels. This paper outlines why the mitochondrion organelle is a suitable biosensor for detecting the early onset of disease; how its use could decrease the cost of health care or lessen the impact of disease; and how a mitochondrion biosensor can be utilized to detect a range of disorders, for which oncology will be presented as an example throughout this article.

## Mitochondrial genomics: a paradigm shift in medicine

The field of mtgenomics, from its inception in 1909 with the discovery of extranuclear inheritance to the completed sequencing of the human mitochondrial genome (mtgenome) in 1981, has been firmly established as a primary arena for the development of genomic biosensors for early cancer detection. Although mtgenomics has been overshadowed in the last two decades by the extraordinary amount of funding and effort directed toward the HGP, it is a more practical backdrop for the discovery and implementation of diagnostic and early detection tools for numerous types of cancers. Historically, cancers such as ovarian, prostate, and colorectal cancer have been very difficult to detect before a devastating prognosis is the result. Utilizing the entire genome of the mitochondrion as a biosensor to detect the onset of these and other diseases is a highly significant medical advancement that could save lives and also makes sound economic sense.

The prospect of discovering countless treatments and possibly even cures for diseases that have a genetic or heritable origin has always surrounded the HGP. Early on genomics was hailed as the beginning of a medical revolution where personalized medicine or health care and treatments tailored to individual genomic profiles would become the norm. However, the enormity and complexity of the human genome, comprised of over 6 billion base pairs, have made this task of developing new medical therapies more complex than originally anticipated by many. What was initially acclaimed as a paradigm shift in modern medicine has become a long process and has necessitated additional programs such as the sequencing of disease specific genomes and characterization of the proteasome as well. Currently, risk assessments based on nuclear genomic data can do little more than provide motivation to encourage diet and lifestyle changes, and since this can be suitably addressed through public wellness campaigns, it becomes hard to justify the expense of offering complete routine genomic evaluations in medicine.

Paradigm shifts are not sudden rearrangements of the world order. They are in fact a series of concepts, theories, and discoveries that gradually build upon one another until enough knowledge accumulates for a larger picture to emerge. The function of all the constituent parts must be understood before an understanding of the overall form can be achieved. When this process of dissection has been completed, in theory, a map should exist that delineates links between each of those parts and their function. How fast these paradigm shifts can occur is directly related to the complexity of the object of study. In stark contrast to the nuclear genome, the human mtgenome is composed of 16,568 base pairs and 37 genes, which by comparison is strikingly simple. If one were to anticipate a paradigm shift towards personalized medicine, particularly where tools for detecting the onset of diseases such as cancer are concerned, a logical place to look would be within the field of mtgenomics. Mathematically, the odds favor a mtgenomic solution. This is suggested by the compactness of the mtgenome and exemplified by mtDNA mutations found in association with numerous diseases, including cancer. A detailed review of these oncologic associations has been presented by Parr et al. in 2007 [2].

mtGenomics is also favored by time since paradigm shifts are not sudden events, but rather the culmination of a series of discoveries and innovations. In 1981, the entire mtgenome was already sequenced, but it would not be until 2006, a full quarter of a century later, when the complete human genome would be sequenced. The field of mtgenomics has had more time to mature, giving scientists substantial opportunities to understand the intricacies of this knowledge base. The benefit of lead time combined with the relative simplicity of the mtgenome and specific biological properties of the mitochondria gives mtgenomics a significant advantage over the larger field of genomics when prospecting for biosensors, especially since mitochondrion organelles can be utilized collectively as a biosensor. From this standpoint, mtgenomics may become an organizational model for the larger body of data that is the HGP.

Table 1[3-8] provides a brief overview of the most significant events in the development of mtgenomics, and for comparison, it includes some of the more recent milestones in the field of genomics.

**Table 1:** Milestones in mitochondrial genomics

**Year**	**Milestone**
1909	Correns and Baur independently identify the first cases of extranuclear inheritance
1934	Goldschmidt suggests that causes for color differences in gypsy moths may reside in the mitochondria
1940	Winge and Laustsen observe ‘inbreeding degeneration’ in diploid yeast which they suggest is due to mitochondrial heredity
1944	Slaughter presents the idea of field cancerization in oral cancer
1949	Ephrussi uses genetic analysis to show that respiration-deficient baker's yeast is due to mutations in the cytoplasm, not the nucleus. Soon after, Slonimski and Ephrussi show the deficiency is due to mitochondrial dysfunction
1953	Slaughter formalizes the concept of field cancerization as preconditioning of tissues for the onset of an ‘as-yet-unknown carcinogenic agent’ which later turns out to be mitochondrial mutation
1963	Nass and Nass use an electron microscope to show that chick embryo mitochondria contain DNA
1964	Schatz et al. shows biochemically that baker's yeast mitochondria contains DNA
Late 1970s	Groundwork for the field of mitochondrial genomics is firmly established
1981	Anderson et al. publishes the sequence and organization of the human mitochondrial genome as 16,569 base pairs long
1990^a^	The US Department of Energy and the National Institute of Health present a 5-year plan to Congress for the Human Genome Project
1999^a^	Chromosome 22 becomes the first human chromosome to be completely sequenced
2000^a^	Working draft of the human genome is completed
2004^a^	Human gene count estimated at 20,000 to 25,000
2006^a^	Human genome completely sequenced in high resolution and is about 3 billion base pairs long

^a^Recent milestones in genomics.

## Mitochondrial genomics and biosensor prospecting

Utilizing mtgenomics as a clinical tool to detect the onset of disease is part of a larger shift towards prevention in medicine. Western medical systems have traditionally focused on treating disease once symptoms have become overt and often acute. This is not a function of intent but a result of the primary diagnostic tools at hand. Instruments like the microscope and stethoscope were the only means of detecting disease available to physicians prior to about 1895 when X‒rays came into use. X‒ray technology made it possible to see into the body without creating a surgical opening; however, its utility was limited primarily to visualizing bones and the outline of dense masses. Until the mid 1950s with the founding of companies like the Thermo Electron Corporation and the subsequent increased access to mass spectrometric instruments, even physiologists primarily conducted nutritional studies by direct physical observation and measurement. However, from this point on, various types of instrumentation began to develop that made numerous types of remote imaging and biochemical analysis possible. This is the era in which mtgenomics came of age. Coinciding with the advent of technology that made it possible to peer into the workings of the human body from many different perspectives, mtgenomics became part of the movement towards developing preventive medicine as opposed to being absorbed into acute care medicine.

There are a number of factors that make mitochondrion organelles well suited for use as a biosensor:

1. Mitochondria contain multiple copies of their own genome, and each cell contains multiple mitochondria, resulting in a 10- to 100-fold copy advantage over nuclear DNA. This means that smaller samples can yield reliable results, and body fluids with low cellularity can be readily evaluated for their mtgenomic content.

2. Mitochondria have their own unique genome which is transmitted clonally through the mother and should remain the same from generation to generation.

3. Mitochondria do not contain histones and are more prone to somatic or spontaneous DNA mutations. The rate of somatic mutation in mtDNA is 10- to 17-fold that of the mutation rate within nuclear DNA. Hence, molecular changes associated with malignant transformations may be detectable much earlier in mtDNA than nuclear DNA.

4. Genomic deletions within mitochondria begin to happen long before traditional histology can identify disease. Biochemical signatures can identify genomic deletions associated with a disease and predict its onset much earlier than a pathologist can observe a problem, thus creating a greater window of time for treatment possibilities.

5. The cancerization field associated with mitochondrial deletions provides a distinct molecular signature that mtgenomic assessment can detect. Though conventional histology of a biopsy sample might not display evidence of malignancy, if the tissue comes from an area that is within the cancerization field, disease onset will be detectable via this signature. Coupling conventional biopsy methodology with mtgenomic assessment to detect this cancerization field provides vital clinical information.

6. The small size of the mtgenome (16,568 bp) places it at a cost advantage compared to the nuclear genome (6,000,000,000 bp) when it comes to analyses. Its compact nature also makes it possible to analyze the entire mtgenome rapidly to identify specific molecular markers with great accuracy.

7. Somatic mutations which affect ATP synthesis by inhibiting OXPHOS also increase the production of reactive oxygen species (ROS) and promote tumor cell proliferation. Detecting these mutations can serve to identify the onset of cancer [9].

8. The mtgenome often displays heteroplasmy, a subset of altered/deleted genomes within the mitochondria. Human tumor tissues that have undergone somatic mutations often display heteroplasmy. Differential rates of heteroplasmy in mitochondria are associated with specific types of tumors and can be used to identify different types of cancer [9].

9. Mutations in the mtgenome have been identified in a wide variety of cancers [2]. Individual as well as combinations of mtgenome deletions can accurately detect cancerization field effects [10].

10. Mitochondria mediate multiple metabolic pathways and are sensitive to physiological changes.

Within a cell, the mitochondrial and nuclear genomes function symbiotically, and their resulting interactions make it possible to detect dysfunction within the cell. Though mitochondria execute various metabolic functions outside of the nucleus of a cell, including synthesizing 95% of cellular metabolic fuel, 1,200 nuclear genes drive and participate in mitochondrial function. While there are 37 genes coded by the mitochondrial genome, 24 of these genes are dedicated to processing 13 genes within the mtgenome that produce protein subunits essential to electron transport. These 13 genes work in concert with 93 nuclear proteins. Some mutations in mitochondrial genes alter the biochemical behavior of the overall mitochondrial/nuclear protein complexes, increasing pools of ROS, which in turn enable tumor growth and may bestow proliferative advantages to the cell [11]. Despite its miniscule size and the small number of genes that comprise the mtgenome, somatic alterations that occur in this molecule directly contribute to tumorigenesis.

There is a deep body of literature describing mitochondrial-nuclear interactions [e.g. [12-14]. The somatic mutations that alter these interactions occur very early in the process of disease onset, even before histopathological changes to cells and tissues are evident to the pathologist. mtGenomic technology makes it possible to exploit these molecular signatures as a biosensor for early indications of disease—detection that would not otherwise be possible for many disorders in the current clinical setting [15]. For example, the sampling error of prostate biopsy is reduced to a 3.4-kb mtgenome deletion which is present in prostate tumor as well as the surrounding normal-appearing tissue [16,17].

**Figure 1:**
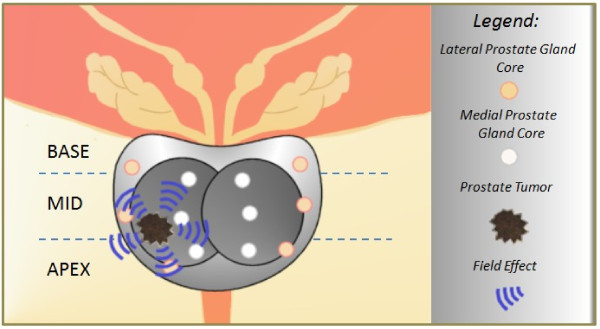
**The cancerization field effect and prostate cancer.** Real-time PCR can be used to identify underlying molecular alterations in normal-appearing tissues, thereby detecting the presence of malignant cells via a cancerization field effect. Though prostate needle biopsy cores may fail to collect a tumor sample, the chemical signature of the tumor cancerization field may be detected in these same samples, optimizing the biopsy procedure and the outcome for the patient [16,17].

There are already mtgenomic tests available for the detection of disease onset, such as Prostate Core Mitomic Test™ (PCMT) offered by Mitomics™ (Thunder Bay, Ontario, Canada). This test, as will other mtgenomic tests, utilizes the cancerization field effect and mtDNA deletions to anticipate the onset and/or presence of hidden or occult cancer. Ideally, this test and others like it act to identify changes in tissue biopsies or other types of biological samples that are indicative of the onset of tumorigenesis. Mitochondrion biosensor tests such as these provide the largest time frame possible to ward off the onset of disease or prevent it altogether (Figure 1).

mtGenomic and complete genomic evaluations will eventually become available with standard medical evaluations and annual physicals. This will provide physicians with comparative longitudinal genomic records and enable the earliest possible routine identification of somatic mutations and the initiation of disease processes. It will also ensure that patients have access to the latest preventative therapies developed in concordance with the field of genomics. Having access to longitudinal genomic records and targeted therapies ascertains the best prognosis for patients. Targeted therapies attack specific types of tissues and are often more tolerable systemically than older chemotherapies which were designed to be aggressive for treating diseases such as cancer identified in acute stages of development.

## Lessening personal and economic costs of disease

Though mtgenomics removes technological barriers for developing early detection biosensor platforms, there are also other considerations to be made. Specifically, developing tools for detecting diseases necessitates the need for early response treatments. This is one of the primary reasons why some of the first mtgenomic tests being developed are for various types of cancer. A number of cancers are difficult to detect using standard medical investigative practices because their symptoms do not become obvious until they reach a very late stage and have become difficult to treat. However, numerous chemopreventive treatments can be used at the earliest stages of these diseases to either suppress or prevent the initiation of carcinogenesis. These treatments are suitable for those deemed at greater risk for developing particular cancers but do not yet have any indications of disease onset, and for those who have begun to display signs of disease, these treatments may halt the progression of precancerous lesions into cancer [18,19].

The National Cancer Institute (NCI) began conducting research on chemopreventive compounds in the early 1980s. To date, about 400 compounds are being actively studied as chemopreventive agents, and more than 40 compounds are being evaluated in a clinical trial setting. Of those agents being studied in a clinical setting, five classes of agents have shown promise, and the NCI considers these classes of compounds to be priority substances for study [20]. Table 2[20,21] briefly summarizes these priority compounds.

**Table 2:** Priority cancer chemopreventive agents

**Class of agent**	**Chemical/brand name**	**Preventive targets**
Selective estrogen receptor modulators and other hormonal agents	Tamoxifen	Breast
	Arzoxifene	Breast
	Acolbifene	Breast
	Raxoxifene/Evista®	Breast
	Finasteride	Prostate
Nonsteroidal anti- inflammatory drugs	Celecoxib/Celebrex®	Colon, breast, prostate, bladder
	Sulindac/Clinoril®	Esophagus, skin
	Aspirin	
Minerals and micronutrients	Calcium compounds	Colon
Glucocorticoids	Budesonide	Lung
Retinoids	All-trans-retinoic acid tretinoin/Retin-A®	Cervix, esophagus, larynx, lung,
	9-cis-Retinoic acid alitretinoin/Panretin®	Nasopharynx, oral cavity, ovary
	13-cis-Retinoic acid isotreninoin/Accutane®	
	4-Hydroxyretinamide, (4-HPR) fenretinide	

Adapted from [21,22].

Toxicity studies can also make use of mtgenomic research. A crucial aspect of evaluating new chemical entities (NCE) for their chemopreventive properties is ensuring that while the compounds disrupt the advance of the disease process, they do not also result in contraindications that are more damaging than the disease itself. A major problem with many new compounds is perturbation of mitochondrial function, resulting in toxic cellular effects. Integrating mitochondrial function studies during the early stages of NCE evaluation provides the opportunity to identify toxic effects from these compounds before they reach later stage development or even human trials. This can save extraordinary amounts of research dollars and time [23].

Additionally, mtgenomic research conducted along with NCE evaluation can stratify the study population into two groups—those that respond to the compound and those that do not. This could keep many drugs from being unnecessarily ejected from the NCE pipeline while also resulting in cleaner, more accurate clinical trial results by removing non‒responders from the study population. This strategy has been used in breast cancer; 5–10% of women are homozygous for low-activity CYP2D6 alleles, and these women cannot properly metabolize Tamoxifen. For women with this genotype, Raloxifene is the chemopreventive therapy of choice. CYP2D6 protein has been indentified and is metabolically active in liver cell mitochondria and functional in drug metabolism. Variants of CYP2D6 alter the efficiency of mitochondrial targeting, suggesting individual responses to drug therapy based on the CYP2D6 protein and mitochondrial interactions [24]. Kelloff et al. [23] summarizes this strategy in the following statement: ‘Molecular Targets that are relevant to risk assessment and patient selection, drug development and outcome evaluation will streamline chemoprevention research. In turn, these molecular targets will lead to effective, safe and personalized cancer prevention strategies for widespread clinical use.’

Moreover, it is of vital importance that drug discovery regimes include comprehensive screening for mitochondrial toxicity to avoid failure in either industry standard or regulatory programs. A classic example of this problem is illustrated by nucleoside reverse transcriptase inhibitors (NRTIs) used to treat HIV infection. NRTIs constrain mtDNA replication resulting in a loss of mitochondria over time, causing liver and muscle toxicity. Other examples include a statin (cerivastatin) and an antidiabetic agent (troglitazone) that have been found to cause muscle toxicity and hepatotoxicity, respectively, due to the negative effects these drugs have on mitochondria [25]. The effect of pharmacological agents on mitochondria is an ongoing concern [26] especially where the majority of individuals benefit while others experience severe contraindications when taking such medications.

mtGenomics has the potential to make meaningful contributions to the area of pharmacogenomics since patients receive obvious benefits from this approach, but making these options available begins with ensuring that the process of disease onset is clearly understood. Michael B. Sporn, former Chief of the Laboratory of Chemoprevention at the NCI and the scientist who coined the term and developed the concept of chemoprevention in the early 1970s, points out that ‘There is still tremendous resistance to the idea of telling people they have early changes in their cells that could someday lead to invasive cancer. The emphasis should be on suppressing carcinogenesis, the development of cancer, before it becomes evident as invasive or metastatic cancer. We need a whole educational mission to get people to think about cancer before they go to the doctor, for example, with a lump in their breast [27].’ For many cancers, the molecular preamble leading to the discovery of a physically observable malignant lesion generally occurs over a 20- to 30-year period. This is a huge window of opportunity for detecting the onset of these diseases. Early detection enables minimally invasive and generally well-tolerated therapies for treatment of these diseases. This means that the impact on quality of life for the patient is minimized to the greatest extent possible.

The economic benefits are also clearly visible. The retail cost for a 30-day supply of Tamoxifen is about $60, and a 5-year supply would cost about $3,700; a 30-day supply of Raloxifene is about $97 with a 5-year supply cost of about $5,800 [28]. Given that in 2006, the first year of treatment for breast cancer in the US cost an average of $30,000 per patient [29,30], utilizing options that prevent the need for any kind of treatment beyond chemoprevention strategies, and monitoring represents a savings to the health care system of more than 80% in the first 12 months alone.

More broadly, figures from the American Cancer Society [19] indicate that the total economic impact of cancer in 2008 was $228.1 billion. Included in this were direct costs of $93.2 billion, losses in productivity amounting to a cost of $18.8 billion, and losses in productivity due to premature death of $116.1 billion. Early screening to detect cancer would significantly reduce losses due to lost productivity attributable to sudden acute health crises for these patients and due to premature death.

Substantial motivation exists for investing in the research and development of early screening methodologies. Multi‒billion-dollar screening markets exist for the top five most prevalent cancers (lung, colon, breast, prostate, and ovarian), ranging from $24 to $401 billion dependent upon the final cost of the tests, which typically range from $100 to $3,500 dollars. Incidentally, current early detection rates for these five types of cancer, though in some instances have improved over what they were even 20 years ago, are still unacceptably low.

## Mitochondrial genomics and human health

While a significant portion of biosensor research has focused on oncology, mtgenomics is applicable to human health in general. Because mitochondria are responsible for converting energy stores into a useable form for our cells, any disorder that affects mitochondrial function could be evaluated using mtgenomics. Mitochondrial function disorders can be caused by mutations or deletions occurring either in the mtgenome or in the nuclear genome (occurring in genes that code for proteins that contribute to mitochondrial function). Mutations in nuclear genes can be inherited from either parent, while heritable mutations in the mtgenome will be transmitted maternally. Heritable mtgenomic mutations can be identified by the presence of heteroplasmy; however, the degree to which these disorders will affect mitochondrial function is generally dependent on the degree of heteroplasmy. If enough copies of unaffected mtDNA are present within a cell, these copies of mtDNA will compensate for the dysfunction of the heteroplasmic copies of mtDNA. Unless individuals with relatively minimal levels of heteroplasmy undergo some sort of trauma or extreme physiological stress that makes it impossible for the unaffected mtDNA to compensate for the heteroplasmic DNA, overt indications of these disorders may never occur. Assessing the degree of heteroplasmy may help these individuals take preventative measures to ensure they minimize the possibility of experiencing such physiological effects.

mtGenomic function may also be affected suddenly by physiological traumas such as hemorrhage or a cardiac event that induces hypoxia and forces mitochondrial function to occur temporarily in an oxygen‒reduced environment. These events may induce mitochondrial damage similar to age‒related declines in mitochondrial function that, as free‒radical theory‒of-aging proponents hypothesize, is due to increased and prolonged exposure to ROS [31]. Other diseases and conditions that increase exposure to ROS and affect circulatory process, such as diabetes mellitus, type II diabetes, and obesity, may also contribute to cumulative disruptions in mitochondrial function. Because the effects of many of these types of disorders accumulate over time, associated somatic mtDNA mutations will also likely accumulate over time. Depending on the degree of severity of these conditions, mitochondrial dysfunction and mtgenomic evidence of dysfunction can occur at any age and ultimately drives the aging process.

Many noncancerous clinical disorders have been associated with mitochondrial dysfunction. Disorders involving the brain and the heart, organs which are high consumers of energy and would suffer from a downturn in mitochondrial production of energy, are notably present in this list. Disorders falling into the neurological category include migraine, strokes, epilepsy, dementia, myopathy, peripheral neuropathy, speech disturbances, and sensorineural deafness. Cardiac disorders include heart failure, heart block, and cardiomyopathy. Gastrointestinal conditions include constipation and irritable bowel syndrome. Respiratory conditions associated with mitochondrial dysfunction include respiratory failure, nocturnal hyperventilation, recurrent aspiration, and pneumonia. Endocrine disorders include diabetes, thyroid disease, parathyroid disease, and ovarian failure. Ophthalmological disorders include optic atrophy, cataracts, and ophthalmoplegia [32]. While this listing is not comprehensive, it shows that the effects of mitochondrial dysfunction are far reaching. While prevalence varies in different populations, research suggests that mtDNA disease is surprisingly high. For example, in a working‒age population (>16 and <60/65 years for female/male patients, respectively) in the north east of England, 9.2 in 100,000 people were found to have manifest mtDNA disease [33].

Presently, mtgenomics is better situated than nuclear genomics for the evaluation of mtDNA disorders due to the smaller size of the mtgenome and number of constituent genes. However, evaluating mtDNA disorders is an area of research where mtgenomics and nuclear genomics could be applied in conjunction since a considerable number of mitochondrial disorders are associated with nuclear genetic inheritance. To exemplify this, the Mitochondria Phenome Knowledgebase cites 174 genes that are associated with 502 different phenotypic features spanning the following categories: oncologic, musculoskeletal, gastrointestinal, respiratory, neurologic, ophthalmologic, genitourinary, cardiovascular, hematologic, dermatologic, metabolic, endocrinologic, immunologic, psychiatric, and numerous miscellaneous categories [34].

While second‒generation technologies have been useful for mtgenomic and nuclear genomic discovery and determining the genetic basis of disease, the rapid action and low cost of genomic sequencing that will become available with third‒generation genomic instrumentation will make it possible to move those discoveries more generally into the clinical setting and widespread medical use [35].

It should not be overlooked that mtgenomics requires careful experimental considerations. Population and group variations in mtgenome sequences may alter or preclude the absence or presence of a specific SNP or deletion(s), leading to incorrect data interpretations for certain populations. Mitochondrial sequencing data must be compared to germ plasma on an individual patient basis if point mutations are to be used as a diagnostic reference. Using published mitochondrial genome standards is inappropriate as these references are not representative of either the individual or the specific disease under study. Moreover, mitochondrial pseudogenes are embedded in the nucleus and often obscure results or lead to inaccurate data interpretation since pseudogene contamination is often mistaken for nucleotide heteroplasmy particularly in sequencing data [36]. This is a noteworthy concern given the sensitivity of next-generation sequencing technology as opposed to di-deoxy chemistry, and extreme care must be taken to preclude co-amplification of nuclear and mitochondrial pseudogenes. Finally, caution is warranted when comparing malignant lesions and normal tissue from the same organ and individual. The field effect is often operative in the normal tissue and may have many mutations in common with the tumor site. Use of normal tissue as a comparative control may preclude reliable results [10].

## Summary and conclusions

Both simplicity of structure and historical timing have placed mtgenomics ahead of the HGP as a technology leader for the development of early detection biosensors for many diseases. Numerous mtgenomic-based tests are currently in development, and some have already entered the marketplace, signaling a transformation in medicine towards prevention as the preferred strategy for treating disease. Early detection biosensors have premiered in oncology, as many types of cancer can be associated with mtDNA deletions. Oncology‒based chemopreventive strategies have been in development since the 1970s and are now available to be paired with early detection strategies. Following closely is the development of biosensors for other mitochondrial disorders. Since these disorders are more frequently hereditary than are many cancers, examining them from the perspective of both mtgenomics and nuclear genomics will provide insight into illness and also serve to develop strategies for dealing with the extraordinary amounts of data generated from the HGP.

Developing mtgenomic‒based technologies presents significant economic benefits for health care management and the development of new drugs. Pairing mtDNA studies with clinical trials stratifies study populations into responders and non‒responders, making the development and usage of these drugs as efficient and effective as possible. Coupling this with the pending release of cheaper and faster third‒generation sequencing technologies creates an opportunity to make mtgenomic‒based early detection biosensors widely available in the near future. This stands to revolutionize the conventional medical experience we are all familiar with into a personalized experience specifically tailored to the well‒being of every individual.

## Competing interests

RP and LM are employed by Mitomics, and RP holds shares in the company.

## Authors’ contributions

RP and LM participated in the design and coordination of this manuscript. Both authors read and approved the final manuscript.
